# Self-Powered Biosensors for Monitoring Human Physiological Changes

**DOI:** 10.3390/bios13020236

**Published:** 2023-02-07

**Authors:** Ziao Xue, Li Wu, Junlin Yuan, Guodong Xu, Yuxiang Wu

**Affiliations:** 1 Department of Health and Physical Education, Jianghan University, Wuhan 430056, China; 2 Beijing Institute of Nanoenergy and Nanosystems, Chinese Academy of Sciences, Beijing 101400, China

**Keywords:** nanogenerator, self-powered, biosensor, health monitoring

## Abstract

Human physiological signals have an important role in the guidance of human health or exercise training and can usually be divided into physical signals (electrical signals, blood pressure, temperature, etc.) and chemical signals (saliva, blood, tears, sweat). With the development and upgrading of biosensors, many sensors for monitoring human signals have appeared. These sensors are characterized by softness and stretching and are self-powered. This article summarizes the progress in self-powered biosensors in the past five years. Most of these biosensors are used as nanogenerators and biofuel batteries to obtain energy. A nanogenerator is a kind of generator that collects energy at the nanoscale. Due to its characteristics, it is very suitable for bioenergy harvesting and sensing of the human body. With the development of biological sensing devices, the combination of nanogenerators and classical sensors so that they can more accurately monitor the physiological state of the human body and provide energy for biosensor devices has played a great role in long-range medical care and sports health. A biofuel cell has a small volume and good biocompatibility. It is a device in which electrochemical reactions convert chemical energy into electrical energy and is mostly used for monitoring chemical signals. This review analyzes different classifications of human signals and different forms of biosensors (implanted and wearable) and summarizes the sources of self-powered biosensor devices. Self-powered biosensor devices based on nanogenerators and biofuel cells are also summarized and presented. Finally, some representative applications of self-powered biosensors based on nanogenerators are introduced.

## 1. Introduction

The human body is metabolizing all day long, and some of the body’s signals can reflect the body’s vital signs and predict its physical state. The monitoring of these body signals plays an important role in disease diagnosis and the health monitoring of the human body [1,2]. Currently, a few instruments are used in the clinical setting to monitor these signals: blood pressure meter, oximeter, electrocardiograph, electromyography, electroencephalograph, thermometer, etc. The disadvantage of such products is that they are large and not easy to carry. Some smart wearable devices are also available on the market, and these devices also have the function of monitoring human signals, such as smart bracelets, smart watches, etc. However, the monitoring accuracy of such products is not good and cannot be used for the diagnosis and auxiliary treatment of diseases.

In order to address the shortcomings and development needs of existing products, researchers have begun to experiment with combining sensors with wearable and implantable technologies and integrating other electronic devices [3]. Supported by the Internet of Things, sensors become nodes that realize a variety of functions, such as in-body sensing, measurement, and data processing and analysis. Due to the characteristics of wearable sensors and implantable sensors, they have the advantages of small size, light weight, functional integration, and good biocompatibility. They can be useful in telemedicine, health monitoring, sports monitoring, etc. [4,5]. Compared to traditional monitoring technologies, sensors provide more accurate and direct access to data and can avoid using private and irrelevant information.

An important research direction for sensors is the improvement and upgrading of materials [6]. Whether implanted in the body or worn on the body surface, these sensors need to be in long-term contact with the body. Therefore, the selection of soft and biocompatible materials is of great importance for the use of sensors on the human body [7]. In addition, motion and external impacts may cause damage to the sensor and the loss of its function. Wearable sensors can be easily replaced, but for sensors implanted in the body, replacement is more problematic. Some self-healing materials are thus good choices for sensors to solve this problem. Some materials can not only restore the mechanical structure after the sensor is cut off but even restore the sensing performance. In order to facilitate a single surgical intervention for implantation without the need for another one for surgical removal, some sensors are biodegradable in vivo, while others can be degraded in the natural environment without polluting it.

With the sustainable development and application of biosensor devices, it has become important to solve the problem of energy supply. Some wearable sensor devices have huge battery sizes [8], which greatly reduces user comfort; for some implanted devices, such as pacemakers, after a few years of use, the power is not high enough for the normal operation of the device. This results in the need for a second surgery to replace the device. This will not only affect the patient’s experience using it but also increase the workload of medical workers. Obviously, the energy supply of the device directly affects the efficiency of the device and the patient’s experience [9], so the improvement of energy supply technology becomes very important. To address the need for the device’s energy supply, researchers have begun to develop self-powered technologies. As the closest toucher of the device, the human body can generate abundant energy, such as a large amount of mechanical energy during movement. Therefore, scientists began to experiment with energy harvesting from the human body and human surroundings. Wang et al. [10] first proposed nanogenerators in 2006 [11,12,13], which collect low-frequency mechanical energy [14]; this makes harvesting energy from the human body and the surrounding environment a reality and has attracted more researchers to study the use of nanogenerators to harvest energy [15]. In addition to nanogenerators, biofuel cells [16] can be used for energy harvesting, which can convert biochemical energy from the human body into electrical energy. In addition to this, there are technologies such as solar cells [17] and electromagnetic generators [18] that can be used as the device’s energy supply, and these technologies have greatly broadened the way in which sensors can be supplied with energy.

This paper first introduces the signal parameters of the human body. Then, it introduces the sensing measurements of wearable and implantable sensors, followed by some sensors that are flexible, self-healing, biocompatible, and degradable. The principles of nanogenerators and biofuel cells are presented in terms of energy supply, as well as some representative work. After that, this paper classifies a large number of sensor devices and finally selects representative work to introduce the application of sensors in human health monitoring. Finally, this paper summarizes the current development status of sensors in the field of human health monitoring and looks at future development directions.

## 2. Human Body Signals

**Figure 1:**
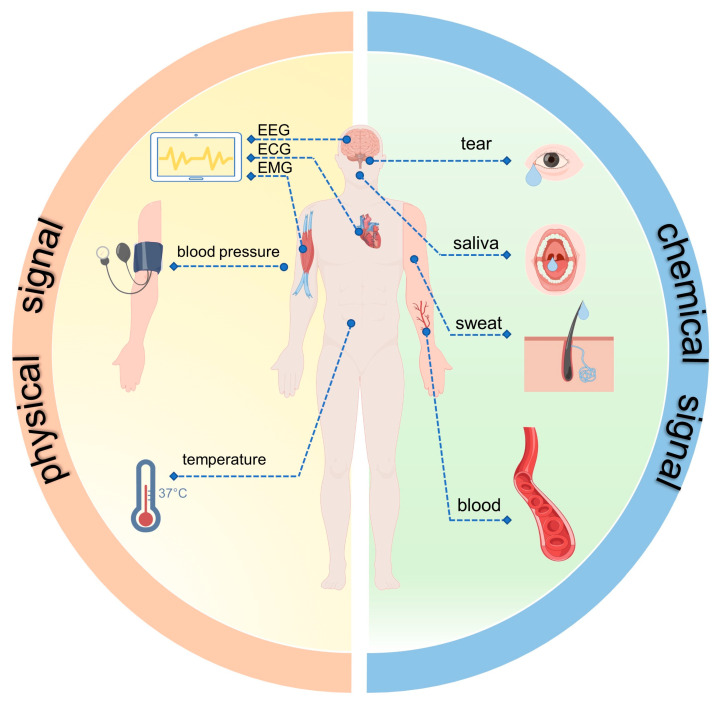
Physical signals (electrical, blood pressure, temperature) and chemical signals (saliva, blood, tears, sweat) that can be collected from the human body. (Figure 1 made by Figdraw).

The human body is rich in physiological signals, and the heartbeat and respiration are continuous even when not in motion. The monitoring of these signals is of great importance for the health monitoring of the human body [19]. Wearable sensors are “eyes” that are developed and manufactured to recognize the various signals that need to be utilized. Sensors made on the basis of different principles can distinguish between different signals. Some sensors are even made to recognize certain signals. Thus, it is particularly important to understand the classification of the types of body signals. Based on the nature of body signals, they can be divided into two categories: physical and chemical signals (Figure 1).

### 2.1. Physical Signals

Physical signals are the physical media used to load information and represent the symbols, states, and signs of the information. The human body is rich in physical signal parameters that can reflect a person’s current state and can even be risk-predictive. These signals mainly include physiological electrical signals [20], blood pressure [21,22], temperature [22,23], respiration [24], and acceleration [25].

Physiological electrical signals. Physiological electrical signals are regular electrical signals closely related to the physiological state of the body during activity or in the resting state [20,26]. These signals are electroencephalographic signals (EEG) [27], electrocardiographic signals (ECG) [28], electromyographic signals (EMG) [29], etc. EEG signals reflect changes in brain activity [30]; ECG signals reflect changes in the activity generated by each heartbeat cycle, which is important for heart health monitoring [31]; and EMG signals reflect the changes that occur during muscle movement and are often used as a guide during exercise training [32].

Blood pressure. Blood pressure is the pressure per unit area of blood vessel wall that blood exerts as it flows through the blood vessels. For patients suffering from hypertension, monitoring blood pressure changes at all times is beneficial. Wearable devices allow for convenient 24/7 blood pressure monitoring [33], increasing the health security of the user.

Temperature. Body temperature reflects, to a large extent, the physical condition of the human body. The temperature of a normal human body is between 36 °C and 37 °C, independent of the surrounding environment. A normal body temperature is necessary for normal metabolism and vital activities. Usually, we feel cold or hot, but the body temperature hardly fluctuates at all. When the body is infected with a disease, the body temperature rises due to the body’s immune system; when the body is exposed to an extremely cold environment, the core body temperature drops and can cause hypothermia [34].

Respiration. Respiration is the process of gas exchange between the organism and the external environment. Respiration can be used as a means of noninvasive health monitoring, and the amplitude and frequency of breathing play an important role in health analysis [4,35]. Wearable sensors can easily and quickly acquire these signals for subsequent monitoring and analysis. By wearing or attaching sensors to the surface of the human body [36], the breathing amplitude and frequency of the human body can be monitored.

Acceleration. Acceleration sensors are mainly used to monitor the speed of human spatial displacement and the speed of movement or changes in human posture. In motion, the speed at which the human body moves is called the displacement speed; the speed at which the human body completes the movement action is called the action speed. Acceleration sensors can monitor the displacement velocity and movement velocity of the human body and identify the posture or state of the human body by changes in acceleration; they can warn of a fall before it is recognized [37] or send a distress signal after it is recognized [38].

Future wearable devices will integrate multiple sensors that can monitor more than one signal [39], and these monitored signals will be used synergistically with each other as an analysis of the user’s health status.

### 2.2. Chemical Signals

Chemical signals, also called biochemical signals, are biochemical molecules that communicate with each other between cells. The human body is also rich in biochemical signals, which can be obtained from body fluids such as blood, saliva, tears, sweat, and urine [40]. Biochemical parameters that can be measured by wearable sensors include pH, lactate, blood glucose, electrolytes, oxygen saturation, etc.

In contrast, saliva, tears, sweat, and urine can be collected in a noninvasive manner, and the biochemical parameters of these fluids can be analyzed to determine the health status of the human body [41,42] and, in some cases, can be used to power the device. In the future, noninvasive collection and monitoring will be the development trend of sensor devices, and electrochemical sensors are particularly suitable for body fluid detection due to their high performance, inherent miniaturization, and low cost. In addition, the fusion of different chemical sensors will allow noninvasive sensors to achieve multi-analyte detection [19,43,44].

## 3. Biosensors in Different Forms and Additional Characteristics

Biosensors can be classified according to different criteria, including their principle, purpose, and function. According to the principle and measurement method of sensors, sensors can be divided into those that monitor physical signals and those that monitor chemical signals; according to the form of sensor contact with the human body, they can be divided into invasive (in vivo) and noninvasive (in vitro), which can also be called wearable sensors and implantable sensors (Table 1).

**Table 1:** Summary of the different forms of biosensors parameters.

Sort	Placement Sites	Target	Range	Sensitivity	Ref.
Wearable	Skin	Muscle deformation	0.5–2.0 V	0.1 V	[45]
	Arm	Volleyball reception	6.65–19.21 kPa	0.3086 V kPa^−1^	[46]
	Wrist	Gesture	\	92.6% accuracy	[47]
Implantable	Aorta (pig)	Blood pressure	0–200 mmHg	14.32 mV/mmHg	[48]
	Left ventricle (pig)/ left atrium (pig)	Endocardial pressure	0–350 mmHg	1.195 mV mmHg^−1^	[49]
	Ligament	Ligament strain	0–600%	25% strain rate	[50]

### 3.1. Wearable and Implantable Biosensors

#### 3.1.1. Wearable Biosensors

Wearable biosensors are noninvasive sensors that acquire signal parameters from the body when attached to the human epidermis. Such sensors are noninvasive to the human body and do not require signal parameters to be acquired through their implantation into the body. Examples include wristbands, belts, watches, bracelets, etc.

**Figure 2:**
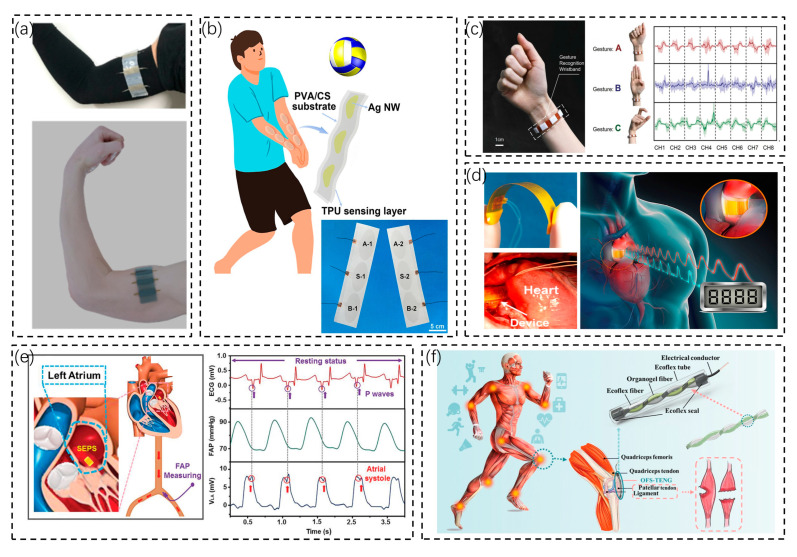
Wearable biosensors and implantable biosensors. (**a**) A stretchable wearable sensor for motion monitoring [45]. Copyright 2019 American Chemical Society. (**b**) A soft and breathable electronic skin that can be worn on the arm for volleyball catch statistics [46]. Copyright 2021 American Chemical Society. (**c**) A wristband for gesture recognition and full keyboard letter input [47]. Copyright 2022 Wiley Online Library. (**d**) An implantable pressure sensor based on a piezoelectric film to monitor blood pressure changes [48]. Copyright 2016, Elsevier. (**e**) An implantable endocardial pressure sensor for monitoring heart rate arrhythmias, ventricular fibrillation, and premature ventricular contractions [49]. Copyright 2019, Wiley Online Library. (**f**) A spiral organogel sensor implanted in the patellar ligament to monitor knee flexion frequency and angle [50]. Copyright 2022 American Chemical Society.

Wearable biosensors can be applied to detect changes in body surface muscle morphology. Wang et al. proposed a stretchable, skin-friendly, and noninvasive wearable sensor for implementing human motion monitoring (Figure 2a) [45]. The deformation of the double-layer silicone rubber structure (DS-TENG) causes friction between the layers due to uneven strain, generating a frictional charge that can be induced in the electrodes as a voltage signal in response to deformation. A 3D muscle sensor made according to this principle can monitor the degree of deformation in different muscle regions based on the change in the output voltage of the DS-TENG. Wearable sensors can be attached to the body surface to monitor stress changes. Wang et al. reported a soft, breathable wearable e-skin (Figure 2b) [46]. This e-skin provided excellent thermal and wet comfort and showed significant antibacterial effects against E. coli and S. aureus. A pressure sensitivity of 0.3086 V kPa^−1^ was measured in the sensing range of 6.65–19.21 kPa. In addition, a 2 × 3 e-skin array-based statistical analysis system for volleyball catching was further developed. This work confirms the practical application of wearable biosensors in the field of sports. Wearable biosensors are also often used for hand posture analysis. Tan et al. developed a gesture recognition wristband with a full keyboard and multiple command inputs (Figure 2c) [47]. A hybrid generator consisting of TENG and PENG was integrated into a small box of 0.4 cm × 0.8 cm × 1 cm. The bracelet monitors hand movements to recognize gestures and can be easily worn on the wrist without interfering with the daily activities of the hand. The bracelet can recognize a variety of gestures with an accuracy of 92.6%. This gesture recognition function can be used for human–computer interaction, for disability assistance, or in special environments and scenarios where verbal communication is not convenient.

#### 3.1.2. Implantable Biosensors

An implantable biosensor is a type of sensor that is implanted in the body to monitor human signal parameters. Due to the characteristics of implantable biosensors, it contacts the body more directly and thoroughly, so it is able to obtain more direct and accurate physiological and biochemical signals. Chen et al. designed a piezoelectric thin film (PETF)-based pressure sensor (Figure 2d) that can be wrapped around the aorta to monitor changes in blood pressure [48]. It was implanted around the ascending aorta of pigs with excellent stability, linearity, and sensitivity (R^2^ = 0.97, sensitivity 14.32 mV/mmHg).

However, implantable sensors also have disadvantages. For people who are afraid of pain, implantable sensors will undoubtedly lead to a bad wearing experience and even aggravate their psychological shadow; for people who are exercising, an invasive method of obtaining physiological and biochemical parameters not only will be inconvenient but also may cause inaccurate data collection because of sweat, the sports environment, dust, and other factors and even cause additional damage. Another drawback of implantable sensors is the energy problem. When using batteries to power the sensor, there is a thermal effect of electrical energy conversion; the batteries also need to be removed for replacement when they run out of power. This is detrimental to the patient experience and expensive. Nanogenerators are a solution to the energy supply of implantable devices. Li et al. reported a miniaturized, flexible, and self-powered endocardial pressure sensor (SEPS) based on the friction nanogenerator (TENG) (Figure 2e) [49]. It can be integrated with a surgical catheter for minimally invasive implantation. The SEPS was implanted in the left ventricle and left atrium of pigs. The SEPS achieved ultra-sensitive real-time monitoring and mechanical stability in pigs, obtaining excellent linearity (R^2^ = 0.997) with a sensitivity of 1.195 mV mmHg^−1^. In addition, the SEPS can detect heart rate arrhythmias, such as ventricular fibrillation and premature ventricular beats, and has an important role in monitoring and diagnosing cardiovascular diseases. The sensitivity of SEPS is 1.195 mV mmHg^−1^. Wang et al. proposed a TENG (OFS-TENG)-based organogel/silica fiber helical sensor with stability and stretchability (Figure 2f) [50]. This sensor can be implanted to monitor real-time strain in ligaments. In vitro and in vivo tests have shown the OFS-TENG to be biocompatible. In the case of the OFS-TENG, it was successfully implanted in the patellar ligament of the rabbit’s knee. When the rabbit flexes its knee, the output is continuously monitored at different frequencies and bending angles. With its good sensitivity and stability, the OFS-TENG offers a broad platform and holds promise for implantable self-powered sensors, wearable devices, and health monitoring.

### 3.2. Biosensors with Additional Characteristics

With the progress in technology and the development of biosensors, they have become smaller and smaller, and their detection accuracy has been greatly improved. In addition, some biosensors have unique properties with the upgrade of materials, for example, flexibility, self-healing, high biocompatibility, and degradability.

**Figure 3:**
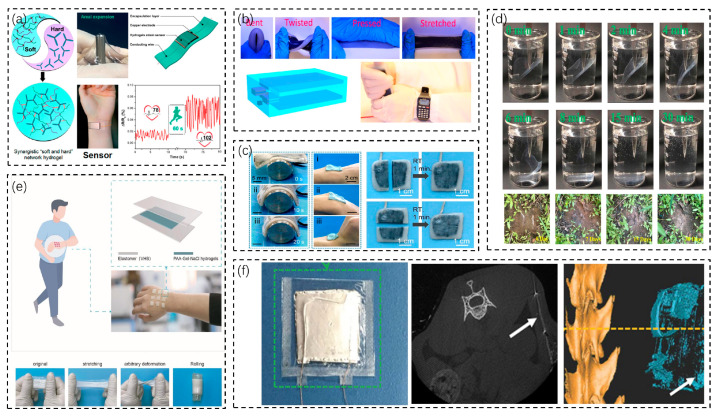
Biosensors with their characteristics. (**a**) A hydrogel sensor that can monitor pulse, with stretchable and self-healing characteristics, and a notch that can self-heal within 5 min after being cut [52]. Copyright 2017, American Chemical Society. (**b**) A stretchable TENG and energy storage composite energy system that can drive an electronic meter with energy derived from the human body [51]. Copyright 2016 American Chemical Society. (**c**) A TENG with shape adaptation that heals itself 1 min after being cut [53]. Copyright 2021, MDPI. (**d**) A biodegradable water-soluble TENG that degrades in water and the natural environment [54]. Copyright 2019, American Chemical Society. (**e**) A self-healing hydrogel that completes self-healing within 2.5 min after being cut at room temperature and can be used as a touch- and pressure-sensing e-skin [55]. Copyright 2016, Science. (**f**) A biodegradable TENG that degrades and is reabsorbed in vivo after completing its task [56]. Copyright 2022, Elsevier.

The good adaptability of flexible wearable and implantable biosensors to the shape of human tissues or organs will improve the performance of the biosensor itself. Wang et al. fabricated a stretchable and fully enclosed self-powered power system by combining a stretchable TENG and a stretchable energy storage system and encapsulating them with flexible materials (Figure 3b) [51]. It is flexible enough to withstand external forces such as stretching and twisting and derives energy from them. The energy obtained from human action when mounted on the human body can drive an electronic watch.

Some wearable biosensors are inevitably bumped and damaged in daily life. It is well known that most organisms in nature have the ability to heal themselves. Some researchers have developed self-healing biosensors that have the ability to heal themselves not only structurally but also functionally. Usually, devices with self-healing capabilities also have soft and stretchable characteristics. The soft, stretchable and self-healing properties of hydrogels can be used to develop new biosensors. Wan et al. prepared a hydrogel with covalent cross-linking and CNCs-Fe^3+^ ligands as binding units in a synergistic soft and hard network structure (Figure 3a) [52]. This hydrogel is robust, stretchable, strain-sensitive, and self-healing. When the hydrogel is cut, the gap can automatically repair itself within 5 min. Based on the properties of the hydrogel, a wearable flexible strain sensor was fabricated that can quickly and accurately monitor changes in finger joints and breathing patterns and also monitor slight pulses in different states of motion. Li et al. produced a self-healing PAA-Gel-NaCl hydrogel, and a repairable single-electrode TENG was fabricated on the basis of the hydrogel for use as an energy-harvesting electronic skin (Figure 3c) [53]. The PAA-Gel-NaCl hydrogel (polyacrylic acid–gelatin–sodium chloride hydrogel) was sliced in half and could self-heal within 2.5 min at room temperature. The resistance of the hydrogel before slicing was 359.28 Ω. After 2.5 min of self-healing, the resistance returned to a stable 363.3 Ω, with only a slight increase over the original state. The hydrogel-based SH-TENG can light up six red light-emitting diodes (LEDs). It also has good sensitivity to low-frequency touch and can be used as an electronic skin for touch and pressure sensing. Wang et al. proposed a TENG that can be shape-adapted and self-healing (Figure 3d) [54]. Using a viscoelastic polymer as the charged material and a CNT-putty composite electrode substrate, SS-TENG is endowed with the ability to adapt to arbitrary irregular surfaces and the instantaneous healing of mechanical damage. SS-TENG can stick to any surface, including adapting to wrist flexion and conforming to wrist motion, and can automatically heal after 1 min of severance.

Generally, implantable biosensors need to be removed after completing their work in vivo, which can exacerbate the pain of patients and also add to the workload of medical personnel, and biodegradable devices are a good way to solve this problem. Wang et al. reported a biodegradable friction nanogenerator (BD-TENG) for short-term in vivo biomechanical energy conversion (Figure 3e) [55]. The BD-TENG consists of a multilayer structure of a biodegradable polymer (BDP) and an absorbable metal. It can be degraded and reabsorbed in vivo after completing its work without any adverse effects. The open-circuit voltage (Voc) of the BD-TENG can reach up to ~40 V, and the corresponding short-circuit current (Isc) can reach ~1 μA.

In addition to biodegradation in vivo, there are devices that can be degraded in the natural environment to reduce the impact on the natural environment. Bian et al. fabricated a water-soluble TENG (WS-TENG) using biodegradable recycled paper and water-soluble graphite electrodes (Figure 3f) [56]. Cellulose nanocrystals (CNCs) were extracted from the paper and further mixed with methylcellulose (MC) to form CNC/MC films, which can be used as a positive electric material with ordinary MC films as a negative electric material to form TENG device materials. The output voltage of the WS-TENG is between 0 V and 2 V, which can accurately distinguish various respiratory states and be used as a real-time signal-monitoring sensor. Additionally, it can degrade in water in 30 min and in the natural environment in 30 days.

## 4. Self-Powered Types of Biosensors

The human body can produce an abundance of energy and is generating energy all the time [57]. Every heartbeat of the human body, every step during movement, and even the heat emitted from the skin or organs can be used as a source for energy harvesting to supply energy to sensor devices. The current technologies for self-powered sensor devices are mainly based on harvesting energy from the human body and human surroundings. The technologies that can be used as self-powered systems are piezoelectric nanogenerators, triboelectric nanogenerators, pyroelectric nanogenerators, and biofuel cells (Table 2).

### 4.1. Piezoelectric Nanogenerators

A piezoelectric nanogenerator [58] (PENG) is a kind of nanogenerator that uses the piezoelectric effect to generate electricity at the nanoscale. The piezoelectric effect is a phenomenon in which some special materials generate an internal electrical potential by an applied external mechanical force. These materials are called piezoelectric materials, such as ZnO. When the piezoelectric material is not subjected to externally applied mechanical forces, the dielectric inside the piezoelectric material is disordered and does not show electrical properties externally. When a mechanical force is applied externally to the piezoelectric material, the dielectric suddenly becomes regular. This process completes the energy conversion of mechanical energy to electrical energy. A PENG has the advantages of low power consumption, simple design, flexibility, and mechanical stability, but its output is relatively low. The output magnitude of a PENG depends mainly on the piezoelectric coefficient and the strain of the piezoelectric material. Therefore, researchers usually increase the interface effect through material compounding or improve the structural design, both of which can increase the piezoelectric performance [59,60].

**Figure 4:**
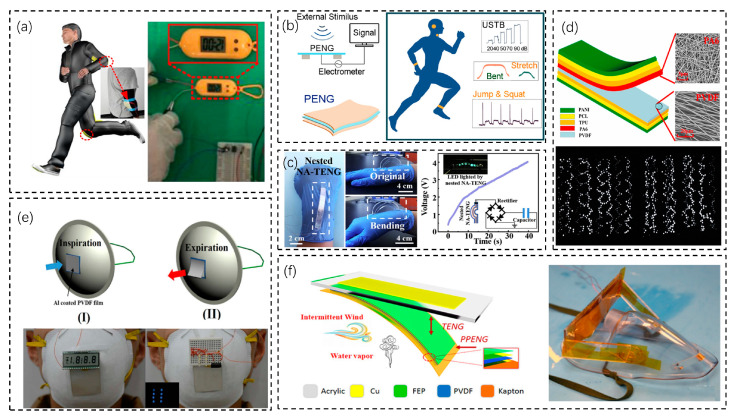
Use of a nanogenerator as an energy supply device. (**a**) A stretchable high-performance PENG that can power an electronic watch [61]. Copyright 2018, Elsevier. (**b**) A highly biocompatible electronic skin powered using a PENG [62]. Copyright 2021, American Chemical Society. (**c**) A nestable arch-shaped flexible TENG that can light up six LED bulbs [69]. Copyright 2020, American Chemical Society. (**d**) A wearable multifunctional TENG with a 10 cm × 10 cm area that can drive 944 LEDs [70]. Copyright 2019, Elsevier. (**e**) A wearable self-powered sensor that uses PyNG for energy supply [75]. Copyright 2017, Elsevier. (**f**) A hybrid nanogenerator that integrates frictional, piezoelectric, and thermoelectric power to harvest energy from wind and water vapor [76]. Copyright 2018, American Chemical Society.

Due to the working principle of the piezoelectric nanogenerator, it can adhere to the human skin to collect the energy generated by the deformation of the skin surface. Chou et al. proposed a high-performance stretchable PENG (Figure 4a) [61]. The use of piezoelectric and conductive particles combined with solid rubber produced a PENG with integrated and high elasticity, excellent piezoelectric properties, and stretchability. This stretchable SPENG can be directly attached to a human joint to achieve kinetic energy harvesting, and the collected energy can drive an electronic meter. Of course, the piezoelectric nanogenerator is also capable of making the device more integrated to achieve simultaneous sensing and energy supply. Wang et al. proposed a highly compatible electronic skin with a PENG energy supply (Figure 4b) [62]. Due to the characteristics of the PENG, the electronic skin can obtain energy while sensing the external world at the same time. Our PENG has a maximum output current density of 9.704 mA·m^−2^ and an open-circuit voltage of ~100 V, capable of powering more than 40 green LEDs or charging a 10 μF commercial capacitor from ~0 V to 6 V for about 500 s.

### 4.2. Triboelectric Nanogenerator

A triboelectric nanogenerator [63,64] (TENG) is a nanogenerator designed according to the frictional electric effect and electrostatic effect. The frictional effect refers to the different electron capture characteristics of different materials: when the surfaces of two materials have different electron capture characteristics of friction, contact, or separation due to external mechanical forces, the two materials will carry different charges, and therefore, when the two materials are in contact, the potential difference between the two material surfaces will induce the flow of electrons from high potential to low potential, leading to the formation of current. Electrostatic induction, also known as electrostatic influence, is the physical phenomenon that the charge in the conductor redistributes without any actual contact with a charged body. When one surface electrode is connected to an external circuit and the two materials are separated by contact, the electrostatic induction phenomenon creates an electrostatic potential that drives the flow of charge and forms an electric current.

The TENG is available in four different operating modes: vertical Contact-Separation Mode, Lateral Sliding Mode, Single-Electrode Mode, and Free-Standing Triboelectric-Layer Mode. The advantages of the vertical Contact-Separation Mode TENG include its simple design and fabrication, high instantaneous power output, and easy multilayer integration. The Lateral Sliding Mode TENG has a lower output but has a wide range of applications in haptics and pressure because one of the friction surfaces does not need to be connected to a wire [65,66,67]. The Free-Standing Triboelectric-Layer Mode TENG does not necessarily need to be in direct contact with the electrode when moving, so there is less wear on the material surface, and it has high durability.

The nanogenerator can realize sensing and energy supply with a special structure. Some specially designed TENGs can obtain energy from the human body’s mechanical energy and obtain human state information from the electrical signal at the same time. This means that the TENG can not only be used as a power supply but also be used directly as an active sensor [68]. Liao et al. proposed a nestable kind of arch-shaped flexible tribological electrical nanogenerator (NA-TENG) (Figure 4c) [69]. It is highly flexible and reproducible and can be attached to the wrist bend to analyze the electrical signal versus bending angle. Its highlight is that it can be assembled like a nesting device, which greatly optimizes the scalability of the NA-TENG’s output performance. They fabricated a three-layer NA-TENG and found that the electrical output improved with the increasing number of layers and the expanding distance between two layers. With changes in materials, triboelectric nanogenerators can find new applications. Yun-Ze Long et al. proposed a multifunctional wearable TENG (Figure 4d) [70]. By using common fabric polymerized polyaniline as an electrode, the sensor is softer and more breathable than the conventional frictional electric structure, so it can be worn for a long time. In the electrical output of the tested TENG, with an area of 4 cm × 8 cm or 10 cm × 10 cm, the short-circuit current and open-circuit voltage output can reach 200 µA and 1000 V, respectively. The 10 cm ×10 cm wearable TENG can drive 944 LEDs.

### 4.3. Pyroelectric Nanogenerators

Pyroelectric nanogenerators [71] (PyNGs) are nanogenerators that harvest energy based on the Seebeck thermal effect using the temperature difference between the two ends of the material. The pyroelectric effect refers to the charge release phenomenon exhibited by the change in polarization intensity with temperature. When the temperature of the material is constant, the polarization intensity of the material does not change, and no current is generated. When the material is heated, the temperature increases, the polarization strength of the material decreases, and a thermal potential is generated. This generated thermal potential drives the carriers from the electrodes to the external circuit. When the temperature decreases, the polarization strength of the material increases, and a current in the opposite direction is generated when the external circuit is connected [72]. Pyroelectric nanogenerators have the advantages of high durability, environmental applicability, and flexibility, and their output size is influenced by the pyroelectric coefficient of the material and temperature changes. They are commonly used in fire alarms, heat sensing, thermal imaging, pollution monitoring, and more [73,74].

Pyroelectric nanogenerators (PyNGs) are mainly used to collect the energy generated by temperature differences, which can be generated by human breathing. Luo et al. proposed a wearable self-powered respiratory sensor using PyNGs for the energy supply (Figure 4e) [75]. PyNGs collect the energy generated by human breathing, and due to the temperature fluctuations caused by human breathing at an ambient temperature of 5 °C, PyNGs can generate output signals with an open-circuit voltage of 42 V and a short-circuit current of 2.5 μA. With a maximum power of 8.31 μW and an external load of 50 MΩ, they can be used to directly drive electronic devices such as LCDs and light-emitting diodes. The electrical signals can be used for harvesting energy from the human body but also reflect the respiratory state and ambient temperature. In addition, different nanogenerators can be coupled together in order to harvest different energies in complex situations. Wang et al. proposed a nanogenerator that integrates a mixture of frictional, pyroelectric, and piezoelectric energy that can efficiently harvest energy from wind and water vapor (Figure 4f) [76]. Each unit of the TENG, PENG, and PPENG (pyroelectric−piezoelectric nanogenerator) can be used to harvest mechanical and thermal energy individually or simultaneously. The hybrid nanogenerator has a higher power output than a single type of nanogenerator. The significance of this is the simultaneous harvesting of different types of energy, which is important in the case of simultaneous changes in airflow and temperature (e.g., respiration).

### 4.4. Biofuel Cell

A biofuel cell (BFC) is a device in which an electrochemical reaction converts chemical energy into electrical energy [77]. A BFC mainly consists of an anode, a cathode, and an electrolyte. The anode oxidizes the biofuel in the presence of a catalyst to generate electrons and protons, the protons pass through the electrolyte to the cathode, and the electrons flow through an external circuit to the cathode. There are many types of bio-cells. The enzymatic biofuel cell (EBFC) is used for biosensing and energy harvesting because of advantages such as good biocompatibility and small size. However, the loading capacity and electron transfer rate of enzymes in biofuel cells are the key factors affecting the output performance of the cells, and researchers usually adopt structural design and material synthesis to improve the cell performance, but it is still difficult to solve the problems of low output power and short service life.

**Figure 5:**
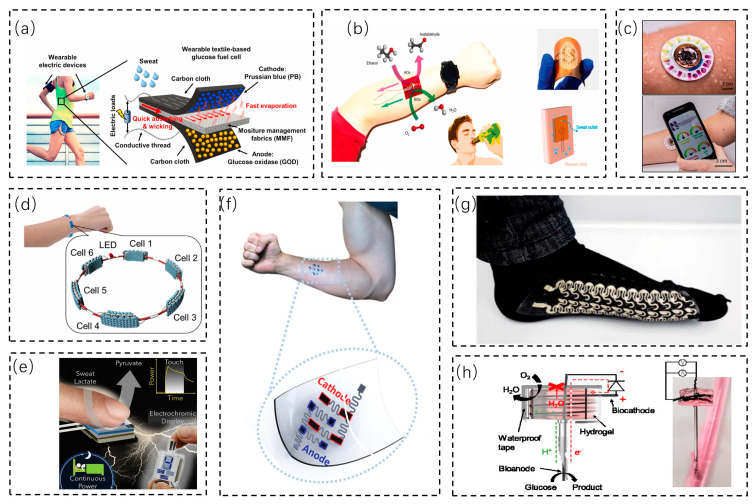
Application scenarios for biofuel cells. (**a**) A fabric-based flexible cell that relies on sweat to generate electricity [78]. Copyright 2020, Elsevier. (**b**) An epidermal biofuel cell that monitors sweat and derives energy from it [83]. Copyright 2021, Elsevier. (**c**) Image of microfluidic patch with embedded sensors and a lactate sensor [82]. Copyright 2019, Science. (**d**) A bracelet with six biofuel cells in series that derives energy from lactic acid in sweat [80]. Copyright 2021, Elsevier. (**e**) A contact biofuel cell that collects energy from lactic acid in sweat [81]. Copyright 2021, Elsevier. (**f**) A stretchable, soft biofuel cell that can be attached to human skin to convert lactic acid from sweat into energy [84]. Copyright 2019, Wiley Online Library. (**g**) A biofuel cell that can be printed onto stretchable, soft fabric with suitable fitting characteristics to harvest energy from sweat [79]. Copyright 2016, Royal Society of Chemistry. (**h**) A needle-shaped biofuel cell that converts glucose in mice into electricity [85]. Copyright 2020, Elsevier.

With the development of biofuel cells and manufacturing technologies, biofuel cells are being integrated into devices and fabrics and have many application scenarios. Biofuel cells combined with fabrics can be fabricated into clothing for more direct contact with the human body. Wang et al. proposed a flexible fabric-based biofuel cell capable of obtaining high power from body fluids through sustainable energy conversion (Figure 5a) [78]. The moisture management fabric (MMF) of sportswear can drive the free flow of molecules. To increase the power output, MMFs were designed and fabricated, and six multi-stack cells were integrated into an armband to supply energy to a sports watch. Wang et al. demonstrated a fabric-based printed BFC that is flexible, stretchable, and stretch-resistant and has suitable fitting characteristics (Figure 5g) [79]. Its close-fitting characteristics make it well suited for harvesting energy from the body surface, and it can capture energy from sweat on the human body surface with a noninvasive energy-harvesting function. The paper shows a sock with an integrated BFC and other sensors, and the real-time signals acquired by the sensors in the sock can be accessed through smartphones and wireless devices.

With the addition of biofuel cells, some devices can easily be self-powered. Yin’s team [80] demonstrated a biofuel cell bracelet that can derive energy from lactic acid in human sweat (Figure 5d). The bracelet uses a hydrophilic fabric to store sweat. By connecting six biofuel cells in series to supply energy, the maximum power output increases from 74 μW for a single BFC to 681 μW for six BFCs, which can drive an electronic watch. This exemplifies the potential of biofuel cells to power wearable electronics. A contact lactate biofuel cell was proposed by Lu et al. (Figure 5e) [81]. Lactate-based biofuel cells (BFCs) rely on the high lactate concentration in human sweat, and epidermal BFCs can easily generate energy by using a lactate oxidase (LOX) bioanode and performing an oxygen reduction reaction (ORR) at the cathode. This biofuel cell can perform energy harvesting even during nighttime sleep without any active movement, and it can be integrated with a piezoelectric generator for further energy harvesting. The energy-harvesting system can be powered quickly to enable the self-powered sensing of sensors. Rogers et al. presented a miniaturized, flexible, battery-free microfluidic electronic system for skin surfaces (Figure 5c) [82]. By combining biofuel cells, colorimetric analysis, NFC electronics, and microfluidics, lactate, glucose, chloride, pH, and the sweat rate/sweat volume can be detected simultaneously. The biofuel cell allows the target analyte to spontaneously generate an electrical signal at the appropriate concentration. The NFC in the assembly allows the portable device to read the electrical signal generated by the biofuel cell. Unlike using a biofuel cell to store energy in the device battery, this is a new idea that can be self-powered.

A biofuel cell patch can be softly fitted to the human skin surface. Sun et al. presented the first example of a flexible wearable epidermal microfluidic human exogenous ethanol/oxygen BFC (Figure 5b) [83]. This biofuel cell can be used in a real-time in situ manner and, through skin abrasion, can sample from the sweat of the drinker and collect continuous power. This epidermal ethanol biofuel cell extends energy acquisition from endogenous to exogenous substances. Chen et al. proposed a stretchable, wearable enzymatic biofuel cell that can harvest energy from sweat (Figure 5f) [84]. The biofuel cell patch has soft and stretchable properties and is attached to the surface of the human body where it is prone to sweat. When the body sweats during exercise, the patch is activated; the lactic acid in sweat is absorbed into the hydrogel electrolyte and permeates the electrodes, and the anode and cathode generate a voltage and current and power the electronic device. In in vitro experiments, the assembled BFCs have a high OCV of 0.74 V and a maximum power density of 520 µW cm^−2^. When applied to a volunteer’s arm, the BFC can generate a maximum power of 450 µW. When connected to a booster, the BFC on the body is capable of powering a light-emitting diode in both pulsed discharge and continuous discharge modes during exercise. However, BFC devices still rely on exercise to produce sweat, which limits their use for non-exercise purposes. The lactate (fuel) sweat concentration and the resulting power may depend on the particular individual and their activity.

Biofuel cells can collect energy from the body surface in addition to converting glucose into electricity in the body. Yin’s team [85] developed an enzyme-mediated needle-shaped biofuel cell that can collect glucose from fruits and mice and convert it directly into electricity (Figure 5h). It can collect 8.5 w from the peritoneal cavity and 16.3 w from the heart in mice. To increase the power from the collected energy and the lifetime of the BFC, the anode needle was coated with an MPC polymer, and the cathode chamber was sealed with waterproof tape. It has the potential to realize energy harvesting from body fluids in the human body to supply energy to wearable devices.

**Table 2:** Summary of the parameters of different types of self-powered biosensors.

Energy Harvester	Materials	Structures	Output	Size	Energy Sources	Ref.
PENG	PZT	Filler-elastomer-based	20 V	5 cm × 4 cm	External deformations	[61]
	PZT-SEBS	Composite elastomer	100 V	3 cm × 3 cm	Stretching	[62]
TENG	Silicone rubber/Al	Contact-separation	1.7 V	5 mm × 10 mm	Finger flexion	[69]
	PANI/PCL	Contact-separation	1000 V	8 cm × 4 cm	friction	[70]
PyNG	PVDF/Al	Three layers	4 2 V	3.5 cm× 3.5 cm	Respiration	[75]
TENG and PyNG	Cu/FEP/PVDF	Contact-separation	20 V (18 m/s)	11.5 cm × 5 cm	Ambient thermal and mechanical energies (wind)	[76]
Biofuel cell	Glucose/carbon fabrics	Textile-based	1.08 V	10 mm × 10 mm (one stack)	Sweat	[78]
	CNT stretchable ink	Textile-based	0.46 V	\	Sweat	[79]
	LOD-based anodic fiber BOD-based cathodic fiber	Textile-based	20 V (6 in series)	2 cm^2^	Sweat	[80]
	Carbon nanotube	BFC-integrated touch-based	0.55 V	1 cm^2^	Sweat	[81]
	CNT	Island–bridge	1.1 mA	\	Sweat	[82]
	PI/PET	Microfluidic module and BFC module	1.01 μWcm^−2^ (45.23 min)	\	Sweat	[83]
	Lactate oxidase and bilirubin oxidase	Island–bridge	0.74 V	0.18 cm^2^	Sweat	[84]
	Enzyme/mediator/carbon nanotube (CNT) composite fibers	Osmium-based polymer/CNT/glucose oxidase/Os-based polymer/CNT.	0.5 V (murine abdominal cavity and heart tissue)	\	Glucose	[85]

## 5. Biosensor Application Demonstration

The daily physiological status of a person can reflect the health status of an individual. Therefore, it is necessary to record these data, which can be used as a basis for analysis and reference. Biosensors can easily measure the physiological data of the human body. Many everyday wearable devices have been designed and developed and put on the market, such as smart bracelets and watches, and usually, watches and bracelets have the function of measuring the heart rate and steps. In addition to these products, biosensors can also be used in different applications for monitoring human physiological information.

**Figure 6:**
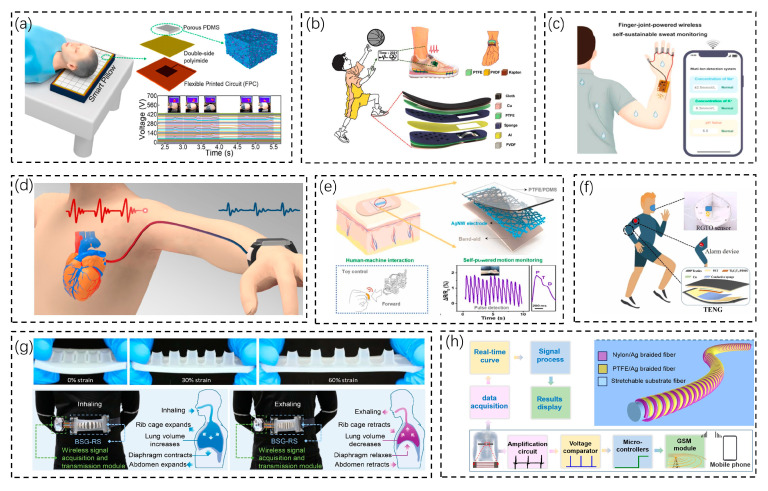
Practical applications of biosensors for human condition monitoring. (**a**) A nanogenerator pillow for monitoring human sleep state [86]. Copyright 2022, American Chemical Society. (**b**) A nanogenerator-based smart insole for gait monitoring and fall warning [87]. Copyright 2022, Wiley Online Library. (**c**) A flexible self-powered sweat sensor that detects Na, K, and pH in sweat [88]. Copyright 2022, Elsevier. (**d**) A nanogenerator-based blood-pressure-monitoring bracelet for monitoring the pulse wave signal of the wearer [89]. Copyright 2022, MDPI. (**e**) A self-powered smart Band-Aid for motion and human–computer interaction [90]. Copyright 2023, Elsevier. (**f**) A breathing alarm device that harvests human motion energy for power sensors [91]. Copyright 2022, Elsevier. (**g**) A multi-pathway respiratory sensor inspired by the gill slit structure of sharks for respiratory monitoring [93]. Copyright 2022, Elsevier. (**h**) A stretchable stress sensor based on the helical structure of TENG for respiratory monitoring for in vivo implantation [94]. Copyright 2022, American Chemical Society.

Sensors can be combined with pillows and footwear to be perfectly integrated into daily living. Wang et al. proposed a smart pillow based on a flexible nanogenerator for monitoring human sleep behavior (Figure 6a) [86]. This flexible and breathable nanogenerator (FB-TENG) is assembled by relying on porous polydimethylsilane (PDMS). The smart pillow is an 8 × 8 self-powered pressure-sensing array laid on a common pillow to achieve real-time monitoring of the static head position and head movement trajectory during sleep, which allows people to fall asleep in a natural way compared to traditional devices for monitoring sleep, such as eye masks and belts. In addition, the smart pillow can also act as a self-powered alarm to serve an anti-fall warning function. The smart pillow can also be expanded in the future to monitor diseases, such as brain diseases and cervical spine diseases. Li Zhou’s group proposed an insole hybrid nanogenerator (IHN) based on the design of a combination of a multilayer friction nanogenerator and an arch piezoelectric nanogenerator (Figure 6b) [87]. It not only converts the mechanical energy of the foot into electrical energy but also distinguishes three motion states: walking, striding, and jumping. Human motion enables a maximum open-circuit voltage of 150 V and a short-circuit current of 4.5 µA. After 8 min of walking, the IHN can charge a 100 µF capacitor to 2.5 V. The energy captured by the IHN is stored in a large capacitor, and when the energy stored in the capacitor reaches a preset high threshold, the capacitor then replaces the battery to provide power. When the energy falls below a preset low threshold, it is powered by the battery again. A self-powered dorsalis pedis artery monitoring system (SAMS) was then also designed to monitor the dorsalis pedis artery pulse signal in real time. This integrated design, which integrates energy collection, storage, and utilization, has important potential in disease monitoring and exercise monitoring and guidance. The above two examples incorporate sensor devices into pillows and insoles to monitor physical signals (posture) of the human body.

In addition to this, everyday wearable products are also a trend of sensor combination, such as electronic patches and bracelets. Sweat is an important source of biochemical indicators. A flexible self-powered sweat sensor was proposed by Chang et al. (Figure 6c) [88]. It detects Na, K, and pH values in sweat and transmits them to the user interface via a built-in Bluetooth module. Biomechanical energy from the human body is collected by the PENG and supplies energy to the device. The device has some drawbacks and still needs improvement: it is not miniaturized or integrated enough in practical applications, which will affect movement to some extent; it needs a PENG to collect energy and then manage and store it before powering the sensors and circuits, and there is a lag in collecting information. In contrast, blood pressure can be monitored in real time by sensors. Tan et al. designed an AI-enhanced blood-pressure-monitoring bracelet/blood pressure predict wristband (BPPW) based on nanogenerator sensing (Figure 6d) [89]. Based on the PENG, the authors designed a mechanical sensor with excellent performance and chose a columnar array microstructure to effectively improve the output performance of the sensor. Its highlight is that after the sensor acquires the pulse wave signal from the wearer, it can be used to predict the wearer’s blood pressure by comparing it with an established artificial intelligence model. The authors used the BPPW on a subject at risk of hypertension for three consecutive days and modeled the prediction of the acquired pulse signal, showing that the predicted values matched the actual situation. A Band-Aid can be applied to the surface of the body, yet it has no sensing function. Sun et al. developed an electronic smart Band-Aid by simply coating a conventional Band-Aid with a network of silver nanowires and a polytetrafluoroethylene/polydimethylsiloxane mixture (Figure 6e) [90]. This smart Band-Aid has excellent frictional electrical properties (VOC = 380 V, ISC = 23 μA, power density = 1.13 Wm^−2^) and strain-sensing properties. It can be used not only for wound protection but also for the self-powered sensing of motion and human–computer interaction. This is a good way to serve the sensing function with a traditional Band-Aid.

For the monitoring of some human signals (e.g., respiration), wearable devices can be used or implantable devices can be chosen. Yu et al. prepared a frictional electric nanogenerator (TENG)-driven self-powered humidity sensor for human respiration monitoring (Figure 6f) [91]. Its highlight is the harvesting of human motion energy (respiration) to power the sensor, which allows real-time respiration monitoring without an external power source. While monitoring humidity, the TENG-RGTO humidity sensor recognizes the state of breathing at different frequencies and intensities in real time. Inspired by nature, bionic sensor systems are leading the development of a new generation of sensor technologies with remarkable features, such as ultra-sensitivity, low power consumption, and self-adaptation [92]. With the help of bionic sensor systems, human signals can be quantified, and machines can be endowed with specific perceptions. Zou et al. proposed a stretchable, multichannel respiratory sensor inspired by the shark gill cleft structure (Figure 6g) [93]. The bionic shark gill (BSG) multichannel nanogenerator can be divided into two layers according to its function: the upper layer is the main functional layer, consisting of several parallel strips of power-gen, and also avoids interference from other factors, which can more accurately identify physiological parameters such as the respiratory rate and respiratory intensity. Respiratory status can reflect the characteristics of certain diseases, which is important for remote-assisted medical diagnosis. By placing the BSG-RS at the waist, the BSG-RS is gradually stretched as the respiratory depth increases and more power-generating units are powered, and the respiratory depth can be estimated synthetically from the respiratory waveform recorded by the multichannel generator. Compared with the traditional single-channel sensor, the multiple channels of BSG-RS can better reflect the changing trend of the respiratory rate recorded by different channels over a period of time. There is also an example of using a wearable sensor to monitor breathing. Ning et al. proposed a Helical Fiber Strain Sensor (HFSS). The HFSS has a helical structure and is wearable (Figure 6h) [94]. Due to its unique helical structure, the HFSS can sense the contraction and relaxation of the thorax and abdomen caused by the heartbeat and respiration and generate electrical signals, even for small stretches of less than 1%. By calculating and processing these electrical signals, the human respiratory rate, exertional lung volume (FVC), exertional expiratory volume in one second (FEV1), and peak expiratory flow rate (PEF) can be obtained. Ning et al. also developed a respiratory monitoring system based on the HFSS, including smart spirometry and smart respiratory alert. Smart lung metering can analyze whether the user has a respiratory disease based on breathing; the smart respiratory alert can automatically call a preset cell phone for help when the user stops breathing for more than 6 s. The above are typical examples of three devices with respiration as an example. In terms of sensing, body deformation due to respiratory movement can be monitored directly from the exhaled airflow, as well as externally. The same is true for energy harvesting. 

## 6. Conclusions

Biosensor devices have rapidly developed in recent years and have advanced or expanded in terms of their energy supply, performance, and application scenarios [95,96]. The biggest limitation in the development of biosensor devices is the energy supply problem: without a continuous and stable energy supply, these devices cannot work properly. With their gradual development, energy supply technologies such as nanogenerators and biofuel cells have shown great promise in the energy supply of biosensor devices, providing a possible future solution for the energy supply of biosensor devices [97]. With the upgrading of materials and the improvement of structures, biosensor devices are also developing from their original huge sizes in favor of miniaturization and low weight. In order to meet comfort requirements for long-term contact with the human body, biosensor devices are flexible [98] so that they fit more closely to the skin or organs; to meet the stability requirements of the device for long-term use, biosensor devices are self-healing and have the ability to recover to their previous state in both form and function; to reduce the impact on the human and natural environments, biosensor devices are highly biocompatible [99] and biodegradable, which means they can be naturally degraded in the human body or in the natural environment. With the development of various classes of biosensor devices, biosensor devices for the acquisition of different signals have been developed, which means that more and more parameters can be recognized by biosensor devices; on the other hand, the accuracy of signal recognition is becoming higher and higher [100], which guarantees more accurate health monitoring and early warnings for the human body. Some biosensor devices made according to human daily life show their future application direction, and it is easy to see that biosensor devices already have the ability to integrate into daily life and perform health monitoring.

## 7. Challenges and Prospects

The advantages and challenges of current biosensors coexist in three main areas: long-term biocompatibility, energy supply, and integration.

For the long-term biocompatibility of biosensors, biosensor devices can be made from flexible materials, making them substantially more comfortable to wear; in addition, the use of some materials makes them self-healing, degradable, and highly biocompatible. There are many receptors that sense internal or external changes on the surface of the body and inside the tissues, and larger implants can be rejected by the body. In order to improve biocompatibility, many researchers have been conducting research for many years to reduce rejection by the body by using biocompatible materials and miniaturizing the device. Future applications to the human body also need to pay more attention to the safety of implantable electronic devices and the stability of devices operating in the body fluid environment.

In terms of energy supply, self-powered devices are already possible with the support of nanogenerators and biofuel cells. However, nanogenerators convert mechanical energy at low frequencies into electrical energy, and the energy-harvesting efficiency of nanogenerators is still at a low level compared to wireless transmission and other methods due to the low frequency of mechanical movements of the human body (e.g., heartbeat and breathing). Moreover, the impedance of the nanogenerator is not consistent with that of the energy storage capacitor, so the energy storage efficiency is not high during the charging process. Improving the efficiency of energy harvesting and the storage of implantable nanogenerators using structural design, material optimization, and engineering techniques is one of the next directions to be studied. Although biofuel cells can also be used for the device’s energy supply, the loading capacity and electron transfer rate of the enzyme are key factors affecting the battery output. Researchers usually improve the structural design and material synthesis to improve the performance of the battery, but it is still more difficult to address the characteristics of low output power and short service life.

Integration is mainly divided into functional integration and integration with daily necessities. In terms of functional integration, biosensors can integrate different types of sensors together, and single devices can achieve multifunctional sensing. The biosensor can also integrate with the data transmission and processing system and analyze the signals collected to achieve the monitoring, analysis, and processing of human physiological signals and thus provide health warnings. In terms of integration with daily necessities, many of the mentioned works have integrated sensors into masks, belts, pillows, watches, and other items. The combination of a biosensor with daily items can make the sensor more suitable for everyday life and lead to inductive and natural sensors. In the era of the interconnection of all things, biosensors can act as Internet of Things nodes and improve the Internet of Things in healthcare.

## Data Availability

Not applicable.
